# Niche Modulation of IGF-1R Signaling: Its Role in Stem Cell Pluripotency, Cancer Reprogramming, and Therapeutic Applications

**DOI:** 10.3389/fcell.2020.625943

**Published:** 2021-01-12

**Authors:** Pei-Chin Chen, Yung-Che Kuo, Cheng-Ming Chuong, Yen-Hua Huang

**Affiliations:** ^1^Department of Education, Taipei Medical University Hospital, Taipei Medical University, Taipei, Taiwan; ^2^Department of Biochemistry and Molecular Cell Biology, School of Medicine, College of Medicine, Taipei Medical University, Taipei, Taiwan; ^3^Department of Internal Medicine, Taipei Medical University Hospital, Taipei Medical University, Taipei, Taiwan; ^4^TMU Research Center of Cell Therapy and Regeneration Medicine, Taipei Medical University, Taipei, Taiwan; ^5^Department of Pathology, Keck School of Medicine, University of Southern California, Los Angeles, CA, United States; ^6^International Ph.D. Program for Cell Therapy and Regeneration Medicine, College of Medicine, Taipei Medical University, Taipei, Taiwan; ^7^Graduate Institute of Medical Sciences, College of Medicine, Taipei Medical University, Taipei, Taiwan; ^8^TMU Research Center of Cancer Translational Medicine, Taipei Medical University, Taipei, Taiwan; ^9^Center for Reproductive Medicine, Taipei Medical University Hospital, Taipei Medical University, Taipei, Taiwan; ^10^Comprehensive Cancer Center of Taipei Medical University, Taipei, Taiwan; ^11^PhD Program for Translational Medicine, College of Medical Science and Technology, Taipei Medical University, Taipei, Taiwan

**Keywords:** IGF-1R, niche, stem cells, cancer stemness, hypoxia, inflammation, extracellular matrix

## Abstract

Stem cells work with their niches harmoniously during development. This concept has been extended to cancer pathology for cancer stem cells (CSCs) or cancer reprogramming. IGF-1R, a classical survival signaling, has been shown to regulate stem cell pluripotency, CSCs, or cancer reprogramming. The mechanism underlying such cell fate determination is unclear. We propose the determination is due to different niches in embryo development and tumor malignancy which modulate the consequences of IGF-1R signaling. Here we highlight the modulations of these niche parameters (hypoxia, inflammation, extracellular matrix), and the targeted stem cells (embryonic stem cells, germline stem cells, and mesenchymal stem cells) and CSCs, with relevance to cancer reprogramming. We organize known interaction between IGF-1R signaling and distinct niches in the double-sided cell fate with emerging trends highlighted. Based on these new insights, we propose that, through targeting IGF-1R signaling modulation, stem cell therapy and cancer stemness treatment can be further explored.

## Introduction

The development of an embryo requires delicate and precise control over cellular mechanisms to establish a structural and functional organism from the stem cells. Recent advances in stem cell research have revealed the detailed processes of embryogenesis, stem cell differentiation, and cell reprogramming. In contrast to embryogenesis, cancer is considered a dysregulated cellular process. Notably, cancer growth use many of the machinery originally used in the development processes, but they are deregulated for cancer growth and metastasis (Reya et al., 2001; Afify and Seno, 2019). Today, the cancer stemness are thought to derive from direct mutations of stem/progenitor cells (cancer stem cell (CSC) model) or the de-differentiation of cancer cells (cancer reprogramming model) (Bjerkvig et al., 2005). Because of their unlimited self-renewal ability and potential plasticity, the cancer cells with stemness properties are also considered responsible for tumor recurrence, drug resistance, and distant metastasis (Clevers, 2011).

Notably, as researchers have continued to explore the essential signaling pathways that regulate stem cell features in stem cells, these pathways have also been found to control cancer initiation, progression as well as the metastasis of CSCs or cancer cells with stemness-related properties (Taipale and Beachy, 2001; Mishra et al., 2005). For example, Wnt signaling is essential for maintaining the structure and homeostasis of intestinal crypts, and its surrounding mesenchymal cells are the major provider of Wnt ligands, which assist in the functional orchestration of intestinal tissue (Mah et al., 2016). Moreover, excessive Wnt signaling activation is the main trigger of colon cancer initiation, and it is highly expressed in colon CSCs (Basu et al., 2016). In addition, transforming growth factor β (TGF-β) signaling plays a major role in regulating self-renewal and pluripotency in stem cells. It was also proven to be susceptible to hijacking by CSCs to maintain cancer stemness (Sakaki-Yumoto et al., 2013). Nevertheless, the underlying mechanism that switches the role of the same signaling-mediated stemness features between stem cell and cancer reprogramming has been less discussed.

**Figure 1:**
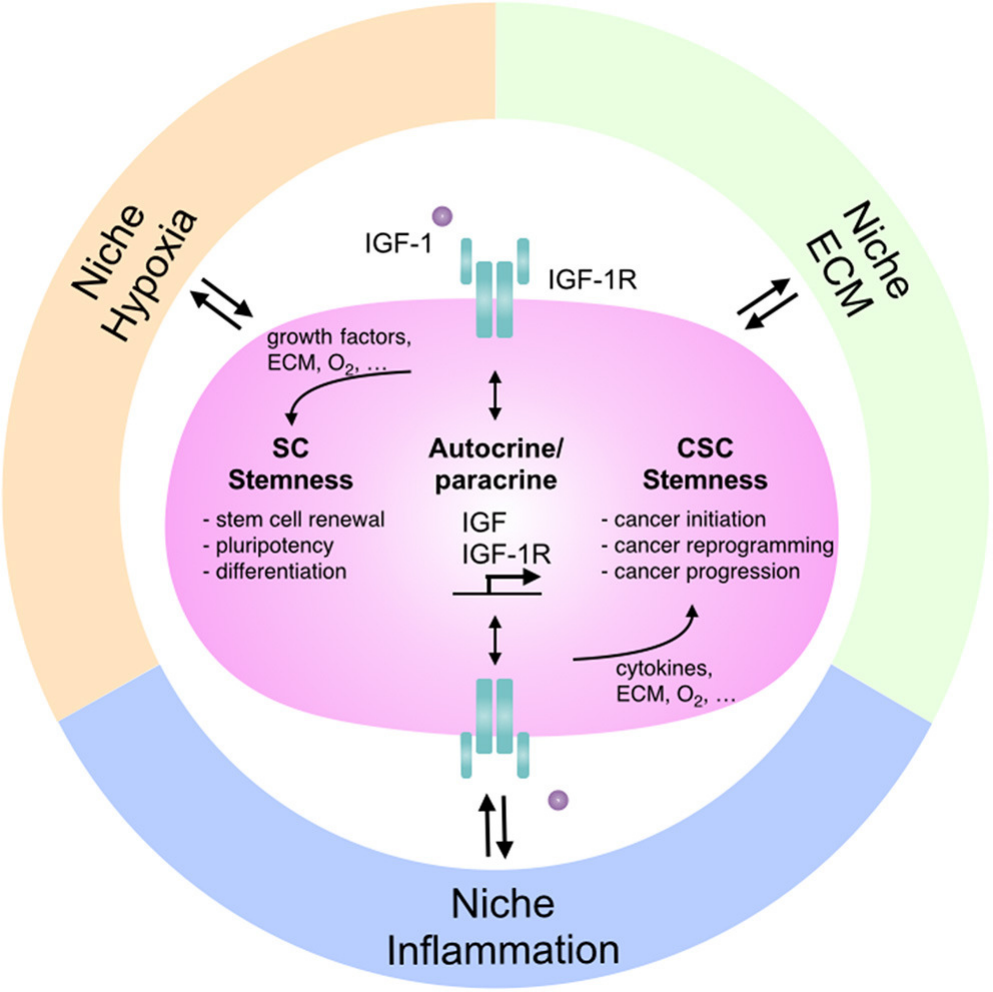
The interactions between IGF-1R signaling and niches: the regulation in stem cells and cancer stemness. Niche microenvironment differs in various physiological and pathological conditions and modulates cellular signaling as well as tissue function accordingly. Different niches affect IGF-1R signaling to regulate stem cell features and control the dual roles of IGF-1R-mediated stemness in stem cell pluripotency and cancer stemness. SC, stem cell; CSC, cancer stem cell; ECM, extracellular matrix.

Insulin-like growth factor-1 receptor (IGF-1R) signaling was recently shown to regulate stem cell characteristics during embryogenesis and tumorigenesis (Chang T.S. et al., 2015; Kuo et al., 2018). Interestingly, the interaction between IGF-1R signaling and the niche environment has been shown to modulate IGF-1R signaling itself and the IGF-1R-mediated cellular responses. Here, we review the interaction between IGF-1R signaling and niche environment with specific focus on niche hypoxia, niche inflammation, and the interaction between cell and extracellular matrix, and further explore how such interaction modulates stemness in various stem cells and CSCs (Figure 1). These insights might provide greater understanding in finding therapeutic opportunities in stem cell therapy and cancer treatment. In the following sections, we target IGF-1R signaling as a basis to explore how key signaling pathways are modulated in diverse conditions as well as the possible switches in controlling the double-bladed role of IGF-1R signaling in stem cells, CSCs, or cancer reprogramming. We also develop a niche modulation concept to explain this conundrum.

## IGF-1R Signaling and Niche Interaction

**Figure 2:**
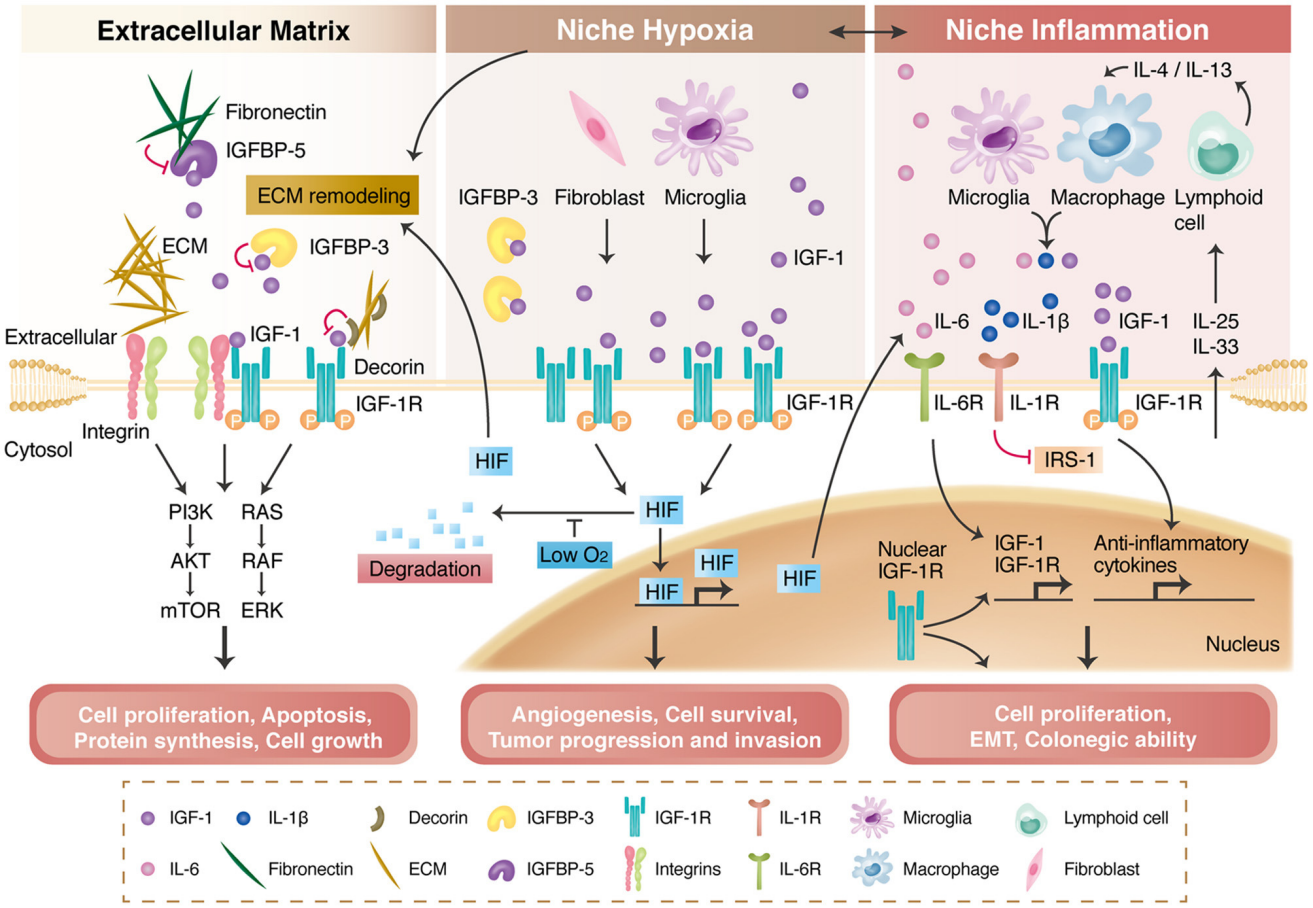
IGF-1R signaling and its modulation by the extracellular matrix (ECM), niche hypoxia, niche inflammation, and nuclear translocation. The ECM and integrins regulate IGF-1R signaling activation through direct binding, transactivation, and the formation of a tertiary complex with IGF-1R. The ECM also interacts with insulin-like growth factor binding proteins (IGFBPs) to modulate IGF-1R signaling (left panel). Niche hypoxia affects IGF-1R signaling by affecting IGFBPs and increasing IGF secretion from niche cells. Hypoxia also stabilizes hypoxia-induced factors (HIFs), enabling HIF-mediated angiogenesis and tumor progression (middle panel). IGF-1R signaling is regulated by niche immune cells in the inflammation process and further modulates local inflammatory responses or tumor progression (right panel). Nuclear-translocated IGF-1R can bind to DNA; can increase *IGF-1R, CYCLIN D1, AXIN2*, and *SNAI2* expression; and is associated with cancer stem cell properties.

IGF-1R is a transmembrane protein in the receptor tyrosine kinase family. It consists of two subunits, namely IGF-1R-α and IGF-1R-β. IGF-1, IGF-2, and insulin are the three ligands of IGF-1R, among which IGF-1 has the highest affinity. Upon ligand stimulation, IGF-1R-β undergoes autophosphorylation and phosphorylates adaptor proteins such as IRS1/2, SHC, and 14-3-3; subsequently, it activates downstream PI3K/AKT, RAS/MAPK, and JAK/STAT signaling, which modulate gene expressions in apoptosis as well as protein synthesis and cell proliferation [reviewed in Girnita et al. (2014)]. Physiologically, IGF-1R signaling is indispensable during normal development. For example, IGF signaling plays a crucial role in regulating organ size. On a large scale, the growth hormone (GH)/IGF-1 axis is essential for postnatal growth. GH insufficiency and deficiency result in smaller body size during an individual's teenage and adult stages; furthermore, IGF-1 expression in GH-deficient mice reverses the decreased body size caused by GH deficiency, suggesting that GH exerts its pro-growth function through IGF-1 (Kaplan and Cohen, 2007; Velloso, 2008). The IGF-1R pathway has also been linked to cancer progression in multiple cancer types, including liver, lung, breast, and colorectal cancers (Dallas et al., 2009; Chang et al., 2013; Chen et al., 2014; Chang T.S. et al., 2015). Given the importance of IGF-1R signaling as a core pathway in regulating multiple cellular responses, more and more studies explore the mechanism of how IGF-1R signaling is modulated. Niche (microenvironment) has been known to modulate cellular signaling through various parameters. The following subsections outline the interaction between different niche properties and IGF-1R signaling and how such interaction affects IGF-1R-mediated cellular responses. We specifically focused on the interaction between IGF-1R and extracellular matrix, niche hypoxia, as well as niche inflammation, and gave a brief review on nuclear IGF-1R, a possible modulator of IGF-1R signaling that was recently reported (Figure 2).

### Niche Extracellular Matrix (ECM) and Integrins

ECM and integrins have been demonstrated to modulate IGF-1R signaling directly through the IGF-1R activation (Girnita et al., 2014). In response to the ECM which contains proteins and glycoproteins, the integrins will induce the intracellular signaling to promote cell proliferation and mobility.

Decorin, an extracellular proteoglycan, shows to bind to both IGF-1 and IGF-1R to repress the IGF-1R activation. Therefore, the downregulation of decorin in bladder cancer results in decreased migration ability and mobility (Iozzo et al., 2011). Mechanical stress and ECM binding enhance the IGF-1/IGF-1R signaling through recruiting integrins and integrin-associated downstream adaptor proteins to the IGF-1R (Tahimic et al., 2016). Supportively, the activation of integrin was shown to transactivate the IGF-1R through adaptor proteins (Girnita et al., 2014). In addition, Takada et al. demonstrated that IGF-1 is able to bind both the IGF-1R and integrins to form a tertiary complex that strengthens the IGF-1R signaling. This observation was further verified by the use of mutant IGF-1, which is defective in integrin-IGF-1R tertiary complex formation, can suppress IGF-1-mediated tumorigenesis *in vivo* in breast cancer and skin squamous cell carcinoma (Fujita et al., 2013; Takada et al., 2017).

Insulin-like growth factor binding proteins (IGFBPs) are proteins secreted by cells to modulate the bioavailability of IGFs [reviewed in Baxter (2014)]. They comprise six main proteins—IGFBP-1 to IGFBP-6—most of which limit the access of IGFs to IGF-1R. IGFBPs participate in various cellular processes, such as cell proliferation, survival, and motility. For example, IGFBP-3 is degraded by matrix metalloproteinase-3 to release IGF-1, activating IGF-1R signaling and cell proliferation (Fowlkes et al., 2004). However, the effect of IGFBP-3 seems to be cell type dependent. In one study, IGFBP-3 exerted a pro-apoptotic effect on doxorubicin-induced endothelial cell death but inhibited apoptosis in serum-starved endothelial cells (Granata et al., 2004). The amount of IGFBPs is also regulated by the ECM. IGFBP-5 was reported to enhance the IGF-1-mediated cell migration of mouse embryonic cells, but this effect was abolished upon fibronectin binding because of the increased proteolysis of IGFBP-5 (Xu et al., 2004).

### Niche Hypoxia

Cells receive diverse signals from extracellular microenvironment and respond accordingly. Among different microenvironment factors, niche hypoxia is one of the most common conditions. Physiologically, hypoxia in the early embryonic stage is crucial in stem cell functions, pluripotency, and organ development (Simon and Keith, 2008; Fathollahipour et al., 2018; Kuo et al., 2018) Pathologically, Eliasz et al. demonstrated that hypoxia-activated Notch 1 can increase IGF-1R expression by binding to the IGF-1R promoter, thereby enhancing IGF-1R-mediated antiapoptosis in lung adenocarcinoma (Eliasz et al., 2010). Moreover, in the pathophysiology of pulmonary hypertension, hypoxic condition will enhance the IGF-1 secretion from the arterial smooth muscle cells, by which to affect pulmonary vessel remodeling (Sun et al., 2016). Meanwhile, it was reported that niche hypoxia in pulmonary hypertension reduces the expression of miR-223 in the lung and right ventricle regions. Decreased expression of miR-223 results in the upregulation of IGF-1R and enhances IGF-1/IGF-1R signaling that mediates right ventricular hypertrophy in the pathophysiology of pulmonary hypertension (Shi et al., 2016).

Niche hypoxia also affects the niche cell interactions, IGFBPs, and exosomes to regulate the IGF-1R signaling. In pancreatic cancer, hypoxic conditions both induce IGF-1 expression in cancer-associated fibroblasts (CAFs) and the IGF-1R expression in pancreatic cancer cells, and such IGF-1R signaling promotes cell mobility (Hirakawa et al., 2016). Furthermore, the secretion of IGF-1 from microglia increases under hypoxia, which eventually enhances VEGFR expression in endothelial cells to promote retinal angiogenesis (Yin et al., 2017). IGFBP-1 phosphorylation is also increased by hypoxia, resulting in limited IGF-1 bioavailability (Shehab et al., 2017). Moreover, hypoxia increases the secretion of IGFBP-3 from cardiomyocytes, which reduces cellular survival through the reduction of IGF-1 bioavailability (Chang R.L. et al., 2015). The exosomes and miRNAs may involve in the underlying mechanism. The miR-29a targeted by exosomal circHIPK3 could upregulate IGF-1 expression in hypoxic endothelial cells, thereby reducing cellular apoptosis (Wang et al., 2019).

### Niche Inflammation

Niche inflammation plays a prominent role in the body's frequent responses to foreign attacks. IGF-1R signaling is involved in the inflammatory responses in the inflammatory microenvironment. For example, IGF-1 is overexpressed in the inflammatory niche of diabetic nephropathy, and the inhibition of IGF-1R relieves both inflammation and fibrosis in diabetic kidneys (Li et al., 2018). In a model of asthma, a well-known inflammatory disease, Shao et al. demonstrated that miR-133a is downregulated in this inflammatory niche and activates IGF-1R signaling, which is responsible for airway remodeling in asthma (Shao et al., 2019). IGF-1R deletion reduces the infiltration of immune cells as well as the expression of inflammation markers in the lung tissues in a murine model of asthma (Pineiro-Hermida et al., 2017). In addition, niche inflammation modulates IGF-1R signaling and affects local cellular responses. Upon allergen exposure, pulmonary epithelial cells activate certain lymphoid cells to secrete interleukin-4 (IL-4) and IL-13, further inducing the secretion of IGF-1 from macrophages. IGF-1 then binds with IGF-1R to reduce the allergic inflammatory response of pulmonary epithelial cells by promoting microvesicle uptake and anti-inflammatory cytokine release (Han et al., 2016). In rheumatic arthritis, an inflammatory disease affecting multiple organs, including the joints and nervous system, increased IL-6 level and number of activated microglia were observed in the serum and hippocampus, respectively (Andersson et al., 2018). Microglia secrete IL-1β in response to an inflammatory environment, and this secretion is associated with increased IGF-1R expression. This correlates with inhibitory IRS phosphorylation in the neurons and impairs IGF-1R-mediated neurogenesis in the hippocampus regions (Andersson et al., 2018). Niche inflammation is also highly correlated with IGF-IR signal activation and cancer formation. For example, in colorectal cancer, tumor growth and niche inflammation are both attenuated by the dual inhibition of IGF-1R signaling and the STAT3 pathway, resulting in much smaller tumor sizes in both primary and metastatic tumors (Sanchez-Lopez et al., 2016).

### Nuclear IGF1R

The traditional concept of membrane receptors has been challenged as an increasing number of studies have suggested that they can be found, with or without their ligands, in cell nuclei. Nuclear IGF-1R was first detected in hamster kidneys and later in cancer cells and highly proliferative non-malignant tissues (Chen and Roy, 1996; Aleksic et al., 2010). After ligand treatment, activated IGF-1R undergoes clathrin-mediated endocytosis before connecting to microtubules and motor protein dynein through the p150^Glued^ subunit of dynactin, which targets nuclei (Aleksic et al., 2010; Packham et al., 2015). As IGF-1R approaches a nucleus, it is sumoylated by RanBP2 before translocating to the nucleus through importin-β/Ran GTPase (Packham et al., 2015). Regarding its function, nuclear IGF-1R possesses a specific DNA binding capacity and seems to be enriched in intergenic regions, suggesting its role in the regulation of gene transcription through the modulation of enhancers as a transcription factor or coactivator (Sehat et al., 2010). Studies have shown that IGF-1R can bind with the transcriptional coactivator of LEF1/TCF to upregulate the gene expression of *cyclin D1* and *axin2* (Warsito et al., 2012) and that it autoregulates its own gene expression (Sarfstein et al., 2012). More surprisingly, nuclear IGF-1R is associated with RNA polymerase II (Aleksic et al., 2010, 2018) and can phosphorylate histone H3 at the Tyr41 position. This modification can stabilize and recruit the chromatin-remodeling protein Brg1 and further induce *SNAI2* expression (Warsito et al., 2016). Physiologically, it was proposed that nuclear IGF-1R might increase cell proliferation while the traditional AKT and ERK pathways promote cell differentiation and survival (Lin et al., 2017). Nuclear IGF-1R in cancers is positively correlated with tumor stage and presents higher protein levels in metastatic cancer (Codony-Servat et al., 2017; Aleksic et al., 2018). High levels of nuclear IGF-1R were detected in PDGFRα^high^/IGF-1R^high^ murine alveolar rhabdomyosarcoma cells and were associated with greater anchorage-independent colony formation ability (Aslam et al., 2013). Furthermore, the amount of nuclear IGF-1R serves as an effective predictor of anti-IGF-1R therapy in sarcoma (Asmane et al., 2012) and of drug resistance in liver cancer cells and colorectal cancers (Bodzin et al., 2012; Codony-Servat et al., 2017). Collectively, nuclear IGF-1R exerts transcriptional regulation activity, and its newly discovered role in cancer provides a novel mechanism in IGF1R-mediated cancer progression and can serve as a new biomarker for predicting clinical prognosis.

## Niche and IGF-1R-Mediated Stemness Expressions in Stem Cells and Cancers

**Figure 3:**
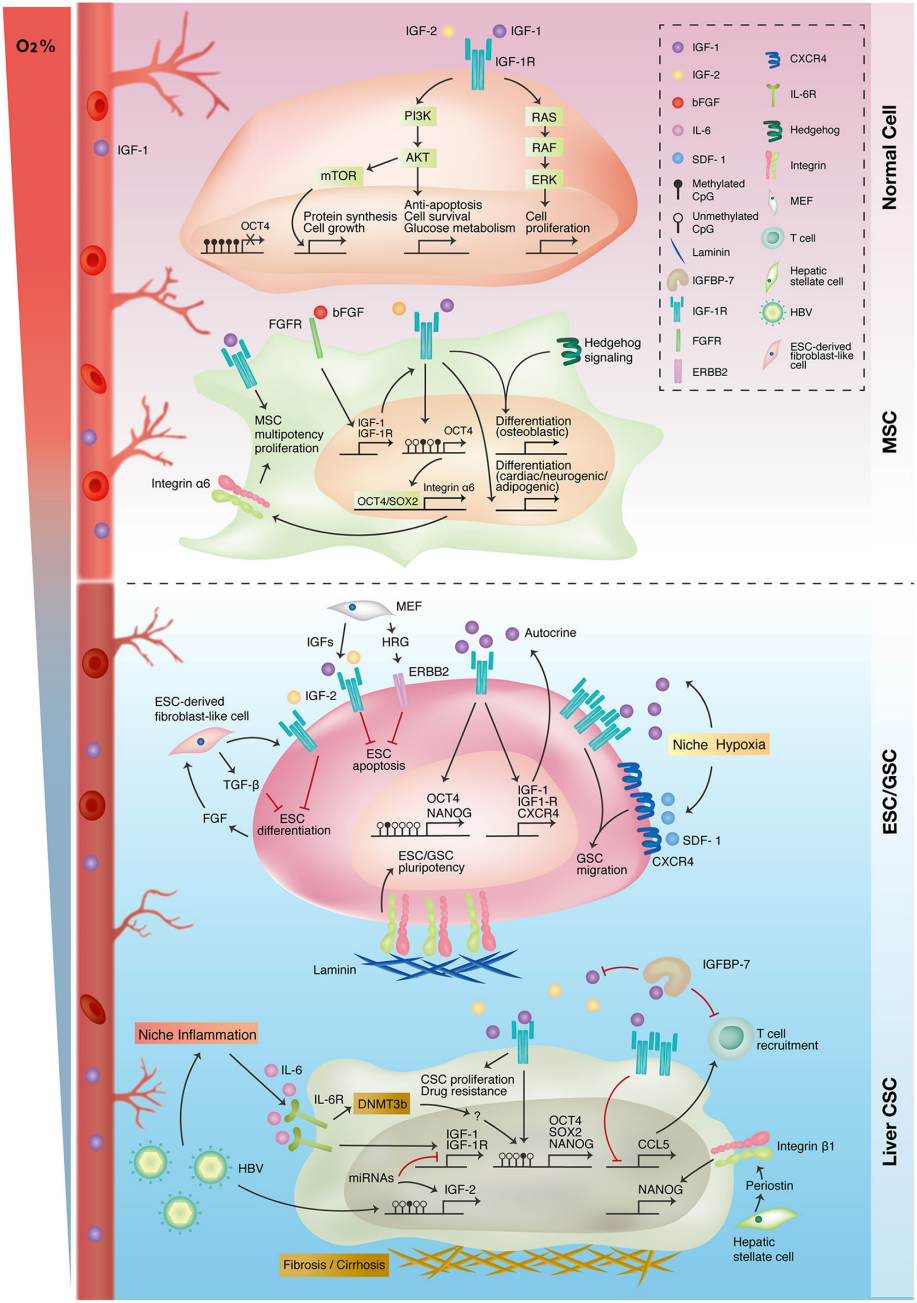
Interplay of IGF-1R-related signaling and niche microenvironment in stemness expressions of different cell types. Traditional IGF-1R signaling promotes cell proliferation, growth, and survival in differentiated normal cells (upper panel). Autocrine IGF-1R signaling promotes mesenchymal stem cell (MSC) proliferation and multiple lineage differentiation through interacting with other signaling pathways (upper-middle panel). In embryonic and germline stem cells (ESC/GSC), niche cells maintain embryonic stem cell survival and pluripotency status through autocrine/paracrine IGF-1R signaling. Niche hypoxia activates both IGF-1R and CXCR4 signaling in promoting the self-renewal and migration of germline stem cells (lower-middle panel). IGF-1R signaling is upregulated in liver cancer because of epigenetic alterations and niche inflammation. The IGF-1R pathway also promotes stemness in liver cancer stem cells (liver CSC) and niche inflammation (lower panel).

The fact that IGF-1R signaling promotes and maintains stemness in both stem cells and CSCs or cancer reprogramming raises the following questions: What transforms IGF-1R signaling from a traditional pathway to being central to the regulation of stem cell properties? Furthermore, how is the dual role of IGF-1R-mediated stemness controlled between the development of embryos and cancers? As mentioned earlier, the interaction between IGF-1R signaling and the niche microenvironment has a crucial role in affecting IGF-1R activation and IGF-1R-mediated cellular responses. Many researches have implicated the importance of niche in controlling stem cell properties, stem cell fate, and lineage differentiation (Morrison and Spradling, 2008; Chacon-Martinez et al., 2018). A similar interplay was also noted between CSCs and cancer niche. We propose that the communication between niche microenvironment and stem cells switches the cell fate into stem cell pluripotency, CSCs, or cancer reprogramming in different stem cells within distinct surrounding niches. Additionally, in normal and malignant cells, interior stimulation could be attributed to the distinct epigenetic signatures of DNA methylation and histone modification at gene promoters. Epigenetic signatures have been proven to change as stem cells differentiate, and the disruption of epigenetic regulation leads to cancer development (Spivakov and Fisher, 2007; Toh et al., 2017). In the following section, we instanced several kinds of stem cell and CSCs to explore how the niche microenvironment modulates the roles of IGF-1R-mediated stemness regulation (Figure 3).

### IGF-1R-Mediated Stemness in Stem Cells

#### Embryonic Stem Cells

ESCs are cells that originate from the inner cell mass and contribute to all cell types of the body. Although many factors have been shown to sustain ESC populations, little is known regarding cell surface receptor activation required for ESC self-renewal. Wang et al. demonstrated that IGF-1R could be activated in human ESCs when the cells were cultured in a mouse embryonic fibroblast (MEF)-derived condition medium (Wang et al., 2007). Furthermore, IGF-1R was coexpressed with the stem cell markers OCT4, SSEA4, and SSEA3 in human ESCs. Blocking IGF-1R activation by using specific antibodies and knocking down IGF-1R in human ESCs limited ESC expansion and induced differentiation (Wang et al., 2007). IGFs, fibroblast growth factor (FGF), and heregulin (HRG) in feeder-, serum-, and KSR-free defined medium are required for the colony formation of human ESCs. Furthermore, human ESCs cultured in this defined media exhibit high resemblance to ESCs cultured in MEF-condition media in terms of global transcriptional expression, micro-RNA expression, and genome methylation profile (Wang et al., 2007). Moreover, IGF/IGF-1R signaling together with HRG/ERBB2 signaling promotes the self-renewal of ESCs and prevents their apoptosis through the PI3K pathway (Wang et al., 2007). Another study of human ESCs also showed that IGF-2/IGF-1R is required for their self-renewal (Bendall et al., 2007). IGF-2 sustains ESC expansion and prevents apoptosis (Bendall et al., 2007). Moreover, the IGF-1R pathway participates in regulating pluripotency in stem cells by promoting and maintaining the expression of pluripotency genes. In mouse ESCs, IGF-1/IGF-1R signaling promotes NANOG expression through the PI3K/AKT pathway, and IGF-1R expression is positively associated with stronger expression of alkaline phosphatase (AP), an undifferentiated ESC marker (Chen and Khillan, 2010). Notably, the promoter of pluripotency genes such as OCT4 and NANOG is unmethylated in ESCs, which might facilitate IGF-1R signaling in promoting pluripotency gene expression (Altun et al., 2010). ESCs themselves can form a microenvironment comprising colony-forming ESCs and ESC-derived fibroblasts (Bendall et al., 2007). These fibroblasts secreted IGF-2 and TGF-β in response to ESC-secreted FGFs. These results demonstrated that IGF-2/IGF-1R signaling supports ESC self-renewal and prevents their apoptosis and that their interaction with the surrounding microenvironment sustains their pluripotent stem cell status and prevents differentiation.

#### Germline Stem Cells

Mouse germline stem cells are responsible for gamete formation during embryogenesis. Our previous study established a serum-free culture system to investigate the defined component of the pluripotency regulation of pluripotent germline stem cells (Huang et al., 2009). Germline stem cells present integrin α6^+^/AP^+^/OCT4^+^ and are capable of differentiating into hepatocyte- and neuron-like cells as well as forming embryonic chimeras; thus, they were designated as AP^+^ germline stem cells (AP^+^ GSCs). We found that IGF-1R signaling supported the pluripotency gene expression of *OCT4, SOX2*, and *NANOG* in AP^+^ GSCs. Moreover, IGF-1 was secreted by AP^+^ GSCs as well as testicular stromal Leydig cells to form an autocrine and paracrine signaling loop. Notably, laminin enhanced the colony-forming ability of AP^+^ GSCs, indicating the role of the ECM in assisting IGF-1R-mediated stemness.

During embryogenesis, hypoxia is a natural process involved in the development of multiple organs through HIFs. Hypoxia is also an active member of the niche microenvironment in regulating stem cell activities (Mohyeldin et al., 2010). Regarding gamete formation, primordial germ cells, a gamete precursor, migrate from the hindgut to the embryonic genital ridge under a hypoxic environment. Notably, hypoxic conditions enhanced the self-renewal ability and OCT4 expression in mouse GSCs. Studies have revealed that IGF-1/IGF-1R expression is induced and IGF-1R signaling is activated by niche hypoxia to synergistically support the proliferation and OCT4 maintenance of GSCs through the PI3K/AKT/mTOR/HIF-2α pathway (Huang et al., 2009, 2014). Interestingly, niche hypoxia is also essential in supporting the migratory potential of GSCs by promoting SDF1/CXCR4 expression and activation. Of relevance here is that hypoxia-activated IGF-1R signaling not only induces SDF1/CXCR4 expression and activation but also transactivates CXCR4 signaling to collectively enhance hypoxia-mediated GSC migration (Kuo et al., 2018). These findings indicate that during embryogenesis, niche hypoxia governs stem cell features through IGF-1R signaling. Taken together, although the IGF-1R pathway is central to the regulation of the stem cell features of self-renewal and pluripotency, it also supports other cellular processes required for precise and organized embryonic development.

#### Mesenchymal Stem Cells

In addition to its role in pluripotent stem cells, IGF-1R signaling has also been shown to be involved in maintaining the self-renewal ability of multipotent and less potent stem cells. MSCs, as previously mentioned, are multipotent adult stem cells with great potential for differentiating into cells such as adipocytes, osteocytes, and neurons. The IGF-1/IGF-1R autocrine loop induces stem cell proliferation and prevents apoptosis through the AKT/GSK-3β/P70S6K pathway in umbilical cord–derived MSCs (UC-MSCs) (Wang et al., 2018). IGF/IGF-1R is activated by basic FGFs (bFGF) to maintain MSC multipotency, and disruption of the IGF-1R pathway with specific molecular inhibitors reduces OCT4/NANOG/SOX2 expression (Park et al., 2009). OCT4/SOX2 can bind to the promoter region of integrin α6 (also known as CD49f or ITGA6) and enhance its expression, and integrin α6 promotes multipotency and stemness in MSCs through the PI3K/AKT/p53 pathway (Yu et al., 2012).

IGF-1R signaling is shown to regulate the differentiation of MSCs. A comparison of gene expression profiles between bone marrow–derived MSCs (BM-MSCs) and BM-MSC-derived adipocytes revealed that IGF-1R signaling was highly involved during the adipogenic differentiation of BM-MSCs (Xu X. et al., 2016). Supplementation of IGF-1 in adipocyte-induction medium promoted the adipogenesis of BM-MSCs by stimulating peroxisome proliferator-activated receptor-γ (PPAR-γ) expression and lipid accumulation (Scavo et al., 2004). Moreover, blockage of IGF-1R activation resulted in less adipogenesis of human UC-MSCs (Park et al., 2009).

In addition to adipogenesis, IGF-1R signaling partakes in osteogenic differentiation (Shi et al., 2015). The IGF/IGF-1R autocrine loop in human BM-MSCs is activated and upregulated by hedgehog signaling, a well-established pathway that is essential for osteoblast differentiation during embryonic skeletal development. IGF-1R and its downstream AKT/mTOR cascade stabilize hedgehog-induced transcription effector Gli2 protein, thereby enhancing a hedgehog-mediated effect on osteoblast differentiation (Shi et al., 2015). Xian et al. also reported that the osteoclast-mediated bone resorption microenvironment contains IGF-1 and TGF-β during bone remodeling. TGF-β attracts BM-MSCs to the sites of bone remodeling, where IGF-1/IGF-1R signaling induces osteoblast differentiation of cells through the PI3K/AKT/mTOR pathway, which preserves bone mass and skeletal microarchitecture. Therefore, IGF-1R knockdown reduces bone mineral density and causes loss of trabecular bone volume in an osteoblast differentiation factor Osx1-expressing cell (Xian et al., 2012). Some studies have also demonstrated that IGF-1R activation induces the expression of osteo/odontogenic markers (such as *OCN, OSX, DSPP, RUNX2, ALP, COL-1*, and *DMP1*) through the JNK/p38 MAPK pathway in dental pulp–derived MSCs (Liu et al., 2018). Additionally, IGF-1R signaling is essential not only for the induction of neurogenesis in adipose tissue–derived MSCs (AD-MSCs) but also for cardiomyocyte differentiation from BM-MSCs (Ning et al., 2008; Gong et al., 2017).

### IGF-1R-Mediated Stemness in Cancer Stem Cells

IGF-1R signaling had also been demonstrated to regulate the cancer stemness in various cancer stem cell models, such as colorectal cancer and breast cancer (Dallas et al., 2009; Chang et al., 2013). We take lung and liver cancer stem cells as an example to explain our concept of niche-specific effect on IGF-1R-mediated cancer stemness.

#### Liver Cancer Stem Cells

Because studies on embryogenesis have suggested that the niche plays a role in the regulation of stem cell characteristics, we further extended this concept to cancer reprogramming to investigate the role of niche microenvironments in hijacking IGF-1R signaling to promote stemness properties in cancers. IGF/IGF-1R is upregulated in hepatocellular carcinoma (HCC) and is associated with tumorigenesis (Martinez-Quetglas et al., 2016). IGF-1R and FGFR are continuously activated in sorafenib-resistant HCC, and these sorafenib-resistant tumor cells exhibit greater tumor-initiating capacity *in vivo* (Tovar et al., 2017). IGF-1R induces the Ras/Raf/Erk-kinase cascade and reduces cellular apoptosis in sorafenib resistance. IGF-1R knockdown in sorafenib-resistant cells increases sorafenib sensitivity, whereas IGF-1R inhibition in naïve cells limits the emergence of drug-resistant cells (Xu Y. et al., 2016).

Emerging evidence has revealed the role of inflammation in carcinogenesis. Chronic inflammation and liver cirrhosis are the predisposing factors in the development of HCC. Hepatitis B virus (HBV) infection was reported to increase the expression of IGF-2 through IGF-1R/MEK/ERK signaling (Ji et al., 2018). Decreases in IGFBP-7 expression increase IGF/IGF-1R signaling, which facilitates the proliferation of HCC and creates a proinflammatory microenvironment (Akiel et al., 2017). IGF-1R inhibition also increases chemokine ligand 5 expression, which is essential for T cell recruitment (Wang et al., 2017). Hepatic stellate cells are responsible for the cirrhotic change and would induce Nanog expression through integrin β1 (Zhang et al., 2017). We previously found that IGF-1R signaling promotes the expression of the pluripotency genes OCT4 and NANOG in hepatitis B virus (HBV^+^)-infected HCC patients. Histological staining of IGF-1R in patients with HBV^+^ HCC is positively correlated with pluripotency gene expression and worse clinical outcomes. Furthermore, expression of OCT4/NANOG in tumor cells is positively correlated with the amount of infiltrated immune cells within the tumor region. Inflammation-condition medium derived from lipopolysaccharide-stimulated HBV^+^ HCC induces the expression of stemness gene OCT4 and NANOG through an IGF-1R-dependent mechanism, suggesting the possible role of active inflammation in controlling IGF-1R-mediated cancer stemness (Chang et al., 2016). Moreover, the serum level of the inflammatory cytokine interleukine-6 (IL-6) is highly elevated in patients with HBV^+^ HCC. Activation of the IL-6/STAT3 pathway induces the expression of IGF-1/IGF-1R, thereby initiating the IGF-1/IGF-1R autocrine and paracrine loop within tumor bulk to induce stemness-related gene expression. Disruption of IGF-1R signaling abolishes IL-6-mediated tumorigenesis *in vitro* and *in vivo*. The coexpression of IL-6, phosphorylated IGF-1R, OCT4, and NANOG corresponds to the worst clinical outcomes for HCC (Chang T.S. et al., 2015).

Epigenetic dysregulation also affects IGF-1R signaling-mediated stemness. IGF-2 is upregulated in HCC due to promoter hypomethylation and higher IGF-2 levels are correlated with the upregulation of hepatic progenitor and angiogenesis markers (Martinez-Quetglas et al., 2016). IGF-1R expression level is modulated by IGF-1R-targeted micro-RNA such as miR122 in HCC (Xu Y. et al., 2016). The concept of epigenetic mechanisms also accords with the re-expression of pluripotency genes in somatic cancer cells. The promoter regions of pluripotency genes OCT4, NANOG, and SOX2 are hypomethylated in HCC compared with normal hepatocytes (Wang et al., 2013). Moreover, niche inflammatory factor IL-6 level positively correlated with IGF-1R, OCT4, and DNA-methyltransferase 3b level in HBV^+^ HCC. This indicates the possible role of epigenetics in controlling IGF-1R-mediated stemness in tumorigenesis under inflammatory niche (Chang T.S. et al., 2015; Lai et al., 2019).

#### Lung Cancer Stem Cells

In lung cancer, IGF-1R signaling regulates CSC activities through the activation of different downstream cascades and cross-talk with the surrounding microenvironment. Xu et al. discovered that IGF-1R expression is highly associated with lung CSC markers CD133 and ALDH1A1 in lung adenocarcinoma. Closer investigation revealed that IGF-1/IGF-1R activates the PI3K/AKT/GSK3β/β-catenin pathway and induces expression of the pluripotency gene OCT4. OCT4 later formed a transcriptional complex with β-catenin that promoted NANOG expression, which is essential for tumorigenesis *in vitro* and *in vivo* for CSCs. Moreover, clinical findings of patient tissue immunostaining have indicated that colocalization of IGF-1R, β-catenin, and OCT4 is strongly correlated with poor prognosis (Xu et al., 2013). IGF-1R also regulates cancer stemness through other downstream pathways. In non–small-cell lung cancer (NSCLC), the IGF-1R/Tescalcin/c-Src complex mediates the STAT3 pathway to induce ALDH1 expression, which preserves CSC features (Lee et al., 2018). In addition, the CD74-NRG1 oncogenic fusion gene activates the PI3K/AKT/NF-κB pathway in lung adenocarcinoma and induces activation of the IGF-2/IGF-1R autocrine loop. Disruption of the IGF-2/IGF-1R circuit abolishes CD74-NRG1-mediated tumor-initiating ability (Murayama et al., 2016). In NSCLC, the IGF-1R pathway is activated by APBB1 and stabilizes SNAIL1 through the PI3K/AKT/GSK3β pathway. This cascade also prevents β-catenin from degradation, thereby promoting the expression of the CSC marker ALDH1 (Lee et al., 2017).

In addition, IGF-1R is regulated by the ECM in controlling epithelial-to-mesenchymal transition (EMT). Fibulin-3 is an extracellular glycoprotein expressed in various tissues and involved in embryonic development (Zhang and Marmorstein, 2010). Suppression of fibulin-3 in lung adenocarcinoma stem cells enhances EMT-associated gene expression. Further data revealed that fibulin-3 competitively inhibits IGF/IGF-1R interaction, and therefore, the loss of fibulin-3 promotes IGF-1R signaling to induce EMT in lung CSCs (Kim I.G. et al., 2014). Chen et al. reported that IGF-2/IGF-1R paracrine signaling between NSCLC and CAFs is essential for sustaining CSCs' features. Fibroblasts isolated from the tumors of patients with NSCLC were used as feeder cells and could maintain an *in vitro* culture system of CSC. IGF-2 secreted from CAFs supported stemness features in CSCs through activation of the IGF-1R/PI3K/AKT pathway, which eventually induced NANOG expression and was responsible for the cells' tumorigenic ability. Depletion of CAFs from the culture system reduced the expression of stemness markers OCT4/SOX2/NANOG and the anchor-independent capacity in CSCs, whereas it increased the expression of differentiation markers such as adenocarcinoma and squamous carcinoma markers (thyroid transcription factor 1, CK7, CK20, p63, and keratin 5/6). Notably, re-coculturing these differentiated cancer cells with CAFs reversed this pattern, suggesting that the microenvironment may be involved in the reprogramming of cancer cells to their CSC state (Chen et al., 2014).

## Targeting IGF-1R Signaling in Stem Cell Therapy and Cancer With Stemness-Related Properties

### IGF-1R Signaling in Stem Cell Therapy

Regenerative medicine provides novel and promising means for recovering organ damage and tissue injury caused by trauma, disease, or age. Because of stem cells' self-renewal ability and pluripotency, they are considered one of the finest tools for treating or preventing diseases and injuries. Stem cells have the ability to interact with and affect adjacent cells and their surrounding microenvironment, forming a niche that might be beneficial for healing and regeneration. For example, emerging evidence shows that stem cells might secrete cytokines and exosomes and play an anti-inflammatory role in injured regions (Vakhshiteh et al., 2019). They may also be recruited to damaged areas by local signals and differentiate into much more mature cells that participate in healing and reconstruction.

One of the most promising possible applications of regenerative medicine is in treating or curing devastating neurological diseases, such as brain stroke, spinal cord injuries, and degenerative neurological diseases. Stroke causes ischemic and hypoxic changes in affected brains. Intravenous injection of human BM-MSCs improved functional outcomes in a rat stroke model of middle cerebral artery occlusion (Zhang et al., 2004). Human MSC administration was detected in ischemic regions, and it increased IGF-1 expression as well as local IGF-1/IGF-1R expression; these increases in expression corresponded with increased cell proliferation and neural progenitor cell recruitment at the damage sites (Zhang et al., 2004). Jeon et al. reported that human BM-MSCs secreted IGFBP-6, which in turn increased extracellular IGF-1 levels in reactive oxygen species (ROS)-induced neuron injury. This IGF-1/IGF-1R signaling protects neural cell death from ROS-mediated toxicity through the PI3K/AKT pathway (Jeon et al., 2017). The survival and migration of neural stem cells were also reported to be regulated by IGF-1R when transplanted into an injured spinal cord, and their survival and migration could be augmented through treadmill training (Hwang et al., 2018). Human dental pulp–derived MSCs with higher expression of IGF-1R exhibited a greater antiapoptotic effect and neural differentiation capacity in a cerebral ischemic rat model. Rats with intracerebral transplantation of IGF-1R^+^ MSCs exhibited improved neurological prognosis and cerebral blood flow (Lee et al., 2016). Additionally, the IGF-1/IGF-1R axis promoted migration potential and neural differentiation in human spinal stem cells without affecting its terminal differentiation. This was used in a phase 1 trial in which human spinal stem cells were injected into cervical, thoracic, and lumbar spinal cord regions to treat amyotrophic lateral sclerosis (ALS), and promising results were reported. Overexpression of IGF-1 in human spinal stem cells enhanced their neuroprotection from excitotoxicity, which enhances the understanding and future prospects of stem cell therapy in ALS (Lunn et al., 2015).

In addition, cardiovascular diseases such as myocardial infarction may benefit from stem cell therapy. Jackson et al. found that after myocardial infarction, IGF-1R expression was upregulated in the ischemic and paraischemic regions of the heart. They then isolated cardiac stem cells from patients with myocardial infarction and overexpressed IGF-1 in the CD90^+^ cardiac stem cell subset (which is thought to have little effect on myocardial repair) through lentiviral transduction. This IGF-1/IGF-1R signaling in cardiac stem cells promotes stem cell survival and protects surrounding cardiomyocytes from apoptosis after infarction. Intramyocardial transplantation of these IGF-1-overexpressing cardiac stem cells in the infarcted area also preserves stem cells in the damaged region and increases myocardial regeneration without affecting its differentiation into cardiomyocytes, smooth muscle, or endothelial cells (Jackson et al., 2015).

In the muscular-skeletal system, stem cell therapy is widely applied for ligament and muscle sprain as well as osteoarthritis and other muscular-skeletal diseases. In an *in vivo* study of the use of AD-MSCs to treat rotator cuff injury, IGF-1R was found to be highly expressed in transplanted AD-MSCs and associated with myocyte differentiation marker myosin heavy chain expression (Kim S.H. et al., 2014). Furthermore, overexpression of IGF-1 in BM-MSCs overcomes the decreased proliferation and osteogenic potential caused by aging (Chen et al., 2017). Taken together, these findings indicate that IGF-1R signaling is involved in repairing and restoring dysfunctional tissue damage and also participates in stem cell therapy. More solid evidence demonstrating IGF-1R signaling manipulation is warranted to provide enhanced strategies for applying stem cell therapy in regenerative medicine.

### IGF-1R Signaling as a Therapeutic Target of Cancer With Stemness-Related Properties

The IGF-1R pathway has been implicated as participating in the regulation of cell proliferation and antiapoptosis in cancers. This raises the possibility of targeting IGF-1R signaling in cancer treatment. However, despite the promising evidence from preclinical studies and early phase 1 trials, larger clinical trials targeting IGF-1R have experienced great setbacks. Some trials were terminated early due to lack of efficiency, while other completed trials showed no improvement in patient outcomes. Many argued that the failure in the phase 2 and 3 trials was largely due to unselected patients and the lack of studies that actually investigated the patient prognosis based on various biomarkers. Pharmaceutical companies have been closing up their project on IGF-1R and only few drugs are now under clinical evaluation (Table 1) (Beckwith and Yee, 2015; Werner et al., 2019).

**Table 1:** Ongoing clinical trials of IGF-1R inhibitors in cancers.

**Drug**	**Combination therapy**	**Indicated diseases**	**Phase**	**NCT number**	**Status^#^**
Ganitumab (AMG479)	-	Metastatic or recurrent sarcoma	1	NCT04199026	NR
	Combination chemotherapy	Newly diagnosed metastatic Ewing sarcoma	3	NCT02306161	Ac/NR
	Dasatinib	Rhabdomyosarcoma	1/2	NCT03041701	R
	Metformin	Breast cancer	2	NCT01042379	R
	Palbociclib	Ewing sarcoma	2	NCT04129151	R
Cixutumumab (IMC-A12)	Paclitaxel	Metastatic esophageal cancer or gastroesophageal junction cancer	2	NCT01142388	Ac/NR

# *R, recruiting; Ac/NR, active not recruiting; NR, not yet recruiting*.

Another way to target IGF-1R signaling in cancer could be eliminating cancer stemness or CSCs by inhibiting IGF-1R given that the IGF-1R pathway plays a crucial role in supporting stemness activities in cancer cells with stemness-related properties. For example, in colorectal cancer, IGF-1R signaling enriches the side-population cell (cells that are best at effluxing the dyes and have been referred to as CSCs) but not the non-side-population cells, indicating the role of IGF-1R in CSC features. Targeting IGF-1R with figitumumab reduces side population cells and ALDH^+^ populations and also inhibits xenograft tumor growth (Hart et al., 2011). Dual inhibitors targeting IGF-1R and other key signaling pathways represent another approach. The co-inhibition of IGF-1R and EGFR inhibits tumor-initiating potential and increases sensitivity to radiation therapy in pancreatic CSCs (Urtasun et al., 2015). The co-inhibition of IGF-1R and EGFR might also render cancer cells sensitive to other combined therapies. However, as indicated in previous clinical trials, specific biomarkers for selecting suitable patients and precise targeting of IGF-1R in CSCs are required. In support of this, Huang et al. demonstrated that the precise targeting of IGF-1R in sarcomas is feasible through the bioengineering of patient-derived chimeric antigen receptor (CAR)-T cells. The authors designed IGF-1R-targeting CAR-T cells and revealed that they selectively exerted their cytotoxicity against sarcoma cells in localized and disseminated xenografts model and extended overall survival (Huang et al., 2015). Further studies should be conducted to identify definitive biomarkers for clinical use and to enhance specific therapeutic targeting of IGF-1R.

## Conclusion and Perspectives

In this review, we highlight the modulation interplay between IGF-1R signaling and microenvironments as well as how this interaction modulates IGF-1R-mediated stemness in stem cells, CSCs, and cancer reprogramming. The implications are 2-folds. The first is about IGF-1R. Given the consequences of the interaction between IGF-1R signaling and niche microenvironments, we reviewed the importance of IGF-1R signaling in regenerative medicine and cancer treatment in terms of stem cell therapy and therapeutic targets toward the cancers with stemness-related properties. Future research will focus on unraveling how IGF-1R signaling regulates stem cell features and to explore the underlying factors that control the dual consequences of IGF1R-mediated stemness. The second is about niche modulation perspective. We contemplate that niche properties can be modulated by adjusting parameters such as extracellular matrix, oxygen tension, cytokines, in different physiological and pathological conditions. The outcome is mediated through selective hub pathways such as IGF-1R signaling in this case, but can be Wnt/beta-catenin in other cases. It is speculative, but we hope more discussions and future investigations along this line can enrich our understanding of stem cell – niche interactions in the future.

## Author Contributions

P-CC, Y-CK, C-MC, and Y-HH: conception and design, writing, review, and/or revision of the manuscript. Y-HH and C-MC: study supervision. All authors contributed to the article and approved the submitted version.

## Conflict of Interest

The authors declare that the research was conducted in the absence of any commercial or financial relationships that could be construed as a potential conflict of interest.
